# The Influence of Spin-Labeled Fluorene Compounds on the Assembly and Toxicity of the Aβ Peptide

**DOI:** 10.1371/journal.pone.0035443

**Published:** 2012-04-30

**Authors:** Jitka Petrlova, Tamás Kálai, Izumi Maezawa, Robin Altman, Ghimire Harishchandra, Hyun-Seok Hong, Daniel A. Bricarello, Atul N. Parikh, Gary A. Lorigan, Lee-Way Jin, Kálmán Hideg, John C. Voss

**Affiliations:** 1 Department of Biochemistry and Molecular Medicine, University of California Davis, Davis, California, United States of America; 2 Institute of Organic and Medicinal Chemistry, University of Pécs, Pécs, Hungary; 3 Laboratory Medicine, Department of Pathology, M.I.N.D. Institute, Miami University, Oxford, Ohio, United States of America; 4 Department of Chemistry and Biochemistry, Miami University, Oxford, Ohio, United States of America; 5 Department of Applied Science, University of California Davis, Davis, California, United States of America; Thomas Jefferson University, United States of America

## Abstract

**Background:**

The deposition and oligomerization of amyloid β (Aβ) peptide plays a key role in the pathogenesis of Alzheimer's disease (AD). Aβ peptide arises from cleavage of the membrane-associated domain of the amyloid precursor protein (APP) by β and γ secretases. Several lines of evidence point to the soluble Aβ oligomer (AβO) as the primary neurotoxic species in the etiology of AD. Recently, we have demonstrated that a class of fluorene molecules specifically disrupts the AβO species.

**Methodology/Principal Findings:**

To achieve a better understanding of the mechanism of action of this disruptive ability, we extend the application of electron paramagnetic resonance (EPR) spectroscopy of site-directed spin labels in the Aβ peptide to investigate the binding and influence of fluorene compounds on AβO structure and dynamics. In addition, we have synthesized a spin-labeled fluorene (SLF) containing a pyrroline nitroxide group that provides both increased cell protection against AβO toxicity and a route to directly observe the binding of the fluorene to the AβO assembly. We also evaluate the ability of fluorenes to target multiple pathological processes involved in the neurodegenerative cascade, such as their ability to block AβO toxicity, scavenge free radicals and diminish the formation of intracellular AβO species.

**Conclusions:**

Fluorene modified with pyrroline nitroxide may be especially useful in counteracting Aβ peptide toxicity, because they posses both antioxidant properties and the ability to disrupt AβO species.

## Introduction

Alzheimer's disease (AD) is characterized by the deposition of various amyloid β (Aβ) aggregates forming amyloid in the brain. Evidence from a variety of studies has established that the oligomeric species of Aβ (AβO) carries the greatest toxicity, triggering a variety of downstream effects resulting in neurotoxicity and cognitive deficits [1], [2], [3], [4]. A major impediment to the development of effective anti-Aβ compounds for AD therapy is that essentially 100% of large-molecule drugs and >98% of small-molecule drugs fail to cross the blood-brain barrier (BBB) [5]. Recently [6], we explored a series of compounds based on a highly rigid tricyclic fluorene ring that were developed as amyloid imaging agents [7]. These compounds contain a tertiary amine electron-donating group attached to one aromatic ring and display excellent pharmacokinetics properties and brain bioavailability. In that work, we reported on the ability of two fluorene compounds to disrupt AβO assemblies and reduce Aβ toxicity [6]. These compounds (K01-162 and K01-186) were identified based on their ability to block cell death secondary to intracellular AβO production. Both fluorene compounds bind and destabilize AβO, and are capable of penetrating the brain and reducing the cerebral amyloid burden in APP transgenic mice. Fluorenes therefore have a potential use in AD therapy by targeting AβO toxicity at both intraneuronal and extracellular sites [6], [8].

In AD, accumulating evidence points to oxidative stress as the ultimate downstream component of Aβ-induced toxicity [9], [10]. For example, Aβ increases NMDA receptor activation, and one of the newer drugs for the treatment of AD (Memantine) targets NMDA receptors in order to block glutamate excitotoxicity. Among other pathways, over-stimulation of NMDA receptors activates phospholipase A, leading to elevated arachidonic acid levels, which in turn generates oxygen free radicals and further activation of phospholipases [11]. Thus the excitotoxicity involves a feedback loop that ultimately leads to neuronal self-digestion via increased Ca^2+^ levels, protein breakdown, free radical formation and lipid peroxidation [10]. As shown previously [6], the anti-amyloid fluorenes have antioxidant properties. Furthermore, because nitroxides such as the pyrroline species can cycle within a redox cascade via a relatively stable non-damaging N-oxyl (nitroxyl) radical intermediate [12], [13], compounds carrying this moiety are likely to have the added potential for decreasing oxidative stress and attenuating the damage caused by reactive oxygen species.

In this study, we apply electron paramagnetic resonance (EPR) spectroscopy to a novel fluorene compound containing a pyrroline nitroxide. This spin-labeled fluorene (SLF) exerts similar potency in AβO disruption and protection against AβO-induced toxicity, while also having superior free radical scavenging compared to the model fluorene compounds. Furthermore, the nitroxide moiety provides an intrinsic reporter group that can be probed by EPR spectroscopy, which may provide a sensitive diagnostic tool for *in vivo* detection of Aβ plaques in patients with AD [14]. Thus, in addition to its potential as a novel bifunctional candidate to address AβO toxicity, the SLF compound may also help as a diagnostic and research tool in elucidating fluorene mechanism of action.

## Results and Discussion

### Bifunctional structure of spin-labeled fluorenes

**Figure 1:**
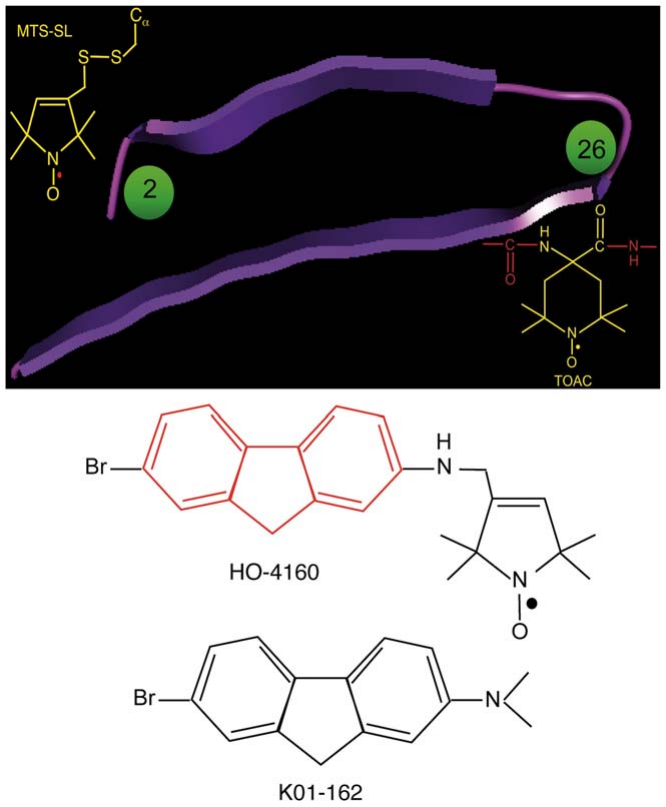
Diagram showing the locations of spin labels in Aβ_(1–40)_, and the structures of the HO-4160 and K01-162 fluorene compounds. Top panel:. the spin labels were targeted to either position 2 (MTS nitroxide spin label) or position 26 (TOAC nitroxide spin label) of Aβ. Position 26 lies within a putative hairpin loop connecting the terminal domains of the peptide, while position 2 is found within the N-terminal domain. Bottom panel: the structure of the SLF compound HO-4160, which is a derivative of the active fluorene K01-162 described in [6]. The core fluorene ring structure is shown in red.

Previously [6], we found the substituted fluorene 7-bromo-2-N,N-dimethylaminofluorene (K01-162) is able to reduce the amyloid burden in mice and block Aβ toxicity in cultured neurons. In addition, K01-162 binds and disaggregates Aβ in its toxic, soluble oligomeric state (AβO). In order to extend the experimental, diagnostic and therapeutic potential of the fluorene agents, the methyl group of dimethylamino was replaced with a pyrroline or piperdine ring to incorporate a spin probe on the fluorene ligand [15]. The structure of spin-labeled fluorene (SLF) HO-4160 (7-bromo-*N*-methyl*-N* –[(2,2,5,5-tetramethyl-2,5-dihydro-1H-pyrrol-3-yl)methyl]-9*H*-fluoren-2-amine radical) is shown in Figure 1.

### SLFs protect against AβO toxicity in cultured neurons

**Figure 2:**
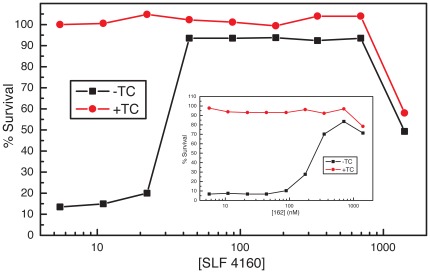
SLF HO-4160 protects against AβO toxicity in cultured neurons. Plotted are the viability values for neuroblastoma MC65 cells with conditional APP-C99 expression as a function of compound concentration. The protective effect against AβO toxicity exceeds that of the previously described base compound, K01-162 (insert).

As demonstrated earlier [6], [8] K01-162 attenuates intracellular AβO accumulation and protects against AβO-induced toxicity in cultured neurons. To test the efficacy of SLFs to block Aβ toxicity in cultured neurons, we examined the potential of a recently described class of SLF molecules to influence the survivability of MC65 neuroblastoma cells [16] containing conditional expression of the C-terminal region (C99) of the amyloid precursor protein (APP). In the MC65 model system, expression of the APP fragment is turned on in the absence of the transgene suppressor, tetracycline (TC). Upon APP-C99 induction, Aβ is generated after proteolysis by cellular γ-secretase [16]. As shown in Figure 2, the viability of cells is severely diminished when the expression of APP-C99 is turned on (−TC), compared to the control cells (+TC) lacking Aβ generation. However, increasing levels of SLF HO-4160 restore cell viability to near control levels, with an EC_50_ of 30 nM. This response is superior to the K01-162 model fluorene (Figure 2, inset). At concentrations above 1 µM, the viability of both APP-induced and un-induced cells declines, reflecting a tolerance limit of the cells to the SLF.

As mentioned above, HO-4160 is one of a group of related SLF compounds that we have recently described [15]. We also measured the ability of the other related SLF analogs (see Fig. S1 in Supporting Information) to block Aβ toxicity. As shown in Table 1, except for compound 5 in this series, each of the compounds offers protection with potency comparable or superior to K01-162. Because HO-4160 provides the greatest amount of protection against Aβ toxicity, we therefore selected this SLF for more detailed analysis of its molecular effects on AβO.

**Table 1:** Potency of SLF compounds [15] against Aβ toxicity as determined by the MTT assay.

Compound	EC_50_ (µM)	Tx_50_ (µM)
3a (HO-4160)	0.03	3.1
3b	0.05	1.4
3b/OH/2HCl	0.1	2.5
3c	0.05	1.3
3d	0.1	2.3
4	0.07	1.6
5	1.2	3.1
K01-162	0.12	10

The structures of 3a and K01-162 are shown in Fig. 1, see Fig. S1 in Supporting Information for other structures.

### SLF HO-4160 reduces AβO accumulation in cultured neurons

**Figure 3:**
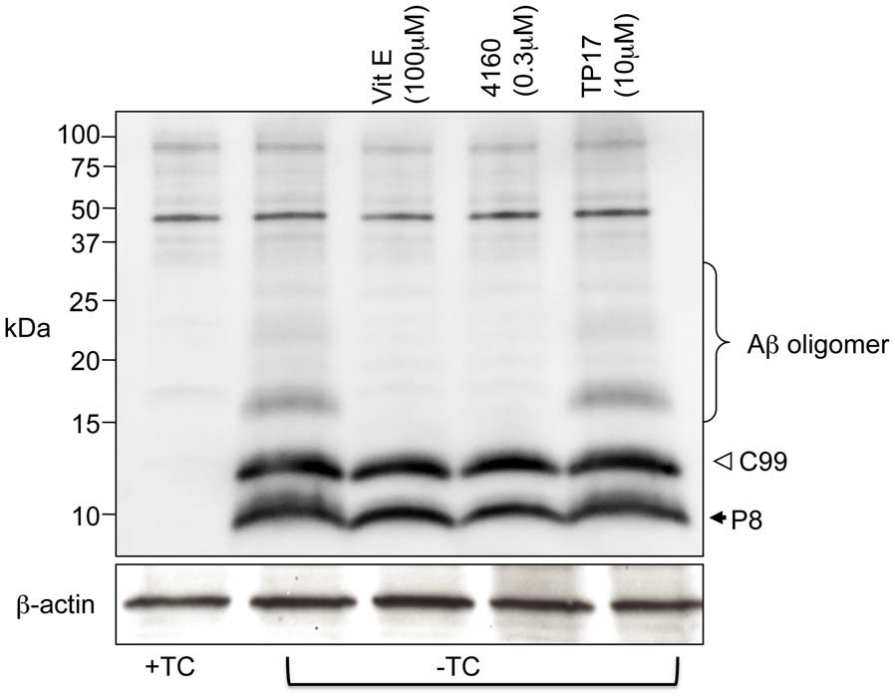
SLF HO-4160 greatly diminishes the intracellular population of oligomeric Aβ Shown are Western blots of extracts from the MC65 human neuroblastoma cell line that conditionally expresses C99, a 99-residue carboxyl terminal fragment of APP in the absence of tetracycline (−TC). C99 is subsequently cleaved by cellular γ-secretase to generate Aβ. TP17, an inactive tricyclic pyrone, serves as a negative control [31], while vitamin E (Vit E), a potent antioxidant that was shown to also block AβO formation in MC65 cells [30], serves as a positive control. p8 is an unresolved band that could be an Aβ homodimer, or a heterodimer of Aβ and APPΔ31 [31], a caspase cleavage product involving residues just downstream from the Aβ origin on APP. The presence of this band does not correlate with MC65 cell death [31]. Blotting was carried out using the Aβ antibody 6E10 (upper panel) and the loading control (lower panel) was probed using an antibody directed against β-actin. The blot shown is representative of 3 replicates.

**Figure 4:**
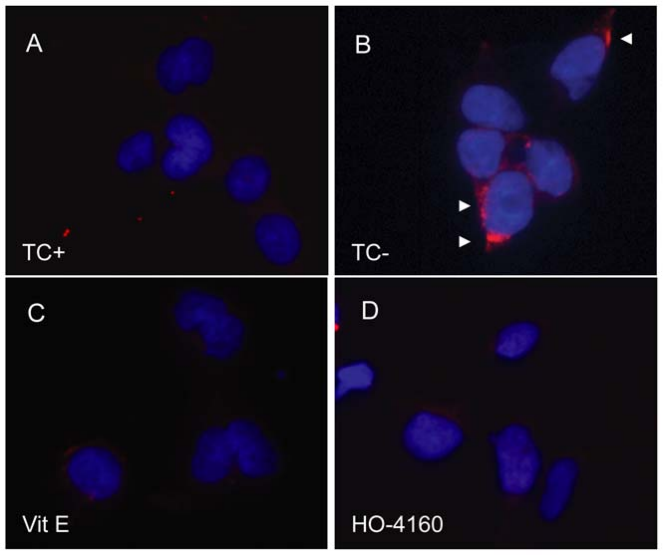
SLF blocks the appearance of oligomeric Aβ species within cultured cells. MC65 cells were treated with the indicated compounds (1 µM) immediately after the removal of the transgene suppressor tetracycline (−TC; panels B–D) to induce AβO accumulation. At 24 hours, cells were fixed, immunofluorescently stained with the oligomer-specific antibody A11 (red) and counterstained with the nuclear dye DAPI (blue). As shown in panel (D), the cytoprotective SLF HO-4160, as well as the antioxidant vitamin E (Vit E) attenuate the accumulation of intracellular AβO (red fluorescent puncta).

Our results clearly demonstrate the protective effect of SLF HO-4160 on MC65 cells expressing APP-C99. To determine whether this protective effect corresponds to a reduction in the AβO burden of the cells, we used both Western blot and immunofluorescent staining to analyze the AβO levels in SLF-treated cells. Western blot analysis of MC65 cellular extracts reveals decreased levels of AβO assemblies of varying sizes in SLF-treated cells expressing APP-C99 (−TC) compared to control cells (+TC). As shown in Figure 3, the Aβ fragments produced by γ-secretase action fail to form any readily apparent oligomers when the cells are treated with 300 nM HO-4160. Complementary results were achieved by using immunofluorescence to probe for AβO using the oligomer-specific antibody A11 [17]. Figure 4 shows greatly decreased levels of AβO assemblies in MC65 cells expressing APP-C99 and treated with SLF (Fig. 4D), compared to untreated cells (Fig. 4B). These results indicate that not only does SLF HO-4160 block the toxic effects associated with Aβ production in cultured neuronal cells, but also directly attenuates the formation of oligomeric Aβ species in the same model.

### Disruption of AβO aggregates by SLF

**Figure 5:**
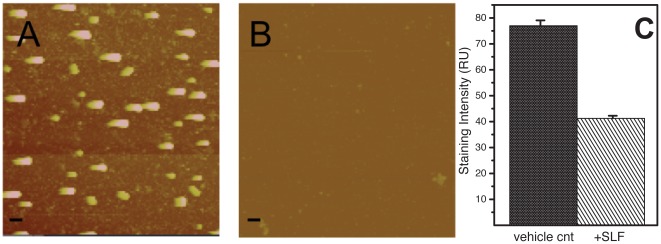
Clearance of AβO aggregates observed by AFM imaging and ThT binding assay. Small oligomers used in this study are formed within 60 minutes (scale bar 10 nm). The direct visualization of oligomers by using AFM revealed the formation of AβO aggregates. (A) 50 µM of Aβ after 24 hours of incubation at room temperature (diameter 5–10 nm). B) 50 µM of Aβ and 50 µM of SLF after 24 hours of incubation at room temperature. (C) Staining for beta-rich assemblies by the amyloid dye thioflavin T for incubations of Aβ with and without SLF HO-4160 as described in Methods. Data are the averages from 3 separate experiments with the error bars representing the SEM.

To test whether SLF HO-4160 blocks the formation of AβO assemblies that can be imaged by atomic force microscopy (AFM) [6], we acquired AFM images of Aβ preparations (50 µM peptide) incubated in PBS buffer for 1 hour, with and without 50 µM SLF treatment. As shown in Figure 5 (A&B), there is a lack of particles >5 nm when Aβ is incubated with the SLF. We also tested whether SLF decreases the formation of particles rich in beta-strand secondary structure using the amyloidophilic dye thioflavin T (ThT). When SLF is included in a 24-hour incubation of Aβ, the ThT fluorescence is decreased by nearly one-half (Fig. 5C). Both the AFM and ThT observations are consistent with a mechanism where the protective activity of HO-4160, like fluorene K01-162, is related to its ability to bind and disaggregate Aβ.

### EPR spectroscopy detects the binding of SLF to Aβ

**Figure 6:**
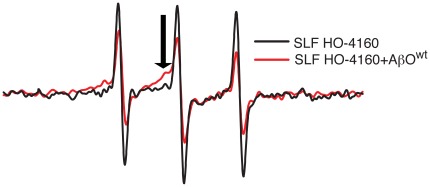
EPR examination indicates the motional freedom of SLF is reduced by interaction with AβO. AβO imparts a broad component (arrow) into the EPR spectrum of HO-4160, reflecting a population of SLF with a slower correlation time.

Due to its small size, the rotational diffusion of the SLF molecule when free in solution will average the hyperfine anisotropy of its EPR spectrum, producing the narrow, time-averaged line shape seen in Figure 6 (black line) indicative of sub-nanosecond diffusion. However, in the presence of AβO, substantial broadening of the SLF (10 µM) signal is apparent (Fig. 6, red line). The most likely cause of the spectral broadening is a reduction in the rate of rotational diffusion by the SLF molecule in solution upon binding to the Aβ peptide. While a small broad peak appears to arise uniquely in the sample containing AβO (see arrow, Fig. 6), we are unable to resolve this feature sufficiently above background. Therefore it is difficult to conclude with certainty whether the resulting spectrum of the SLF in the presence of Aβ represents the compound docked to a mixture of monomers and higher oligomers, or a mixture of bound and unbound SLF. Given the high affinity of fluorenes for AβO [6], we expect that the SLF compound is entirely occupied by the excess Aβ peptide.

### Oligomerization of Aβ as reported by peptide containing the TOAC spin label at position 26

**Figure 7:**
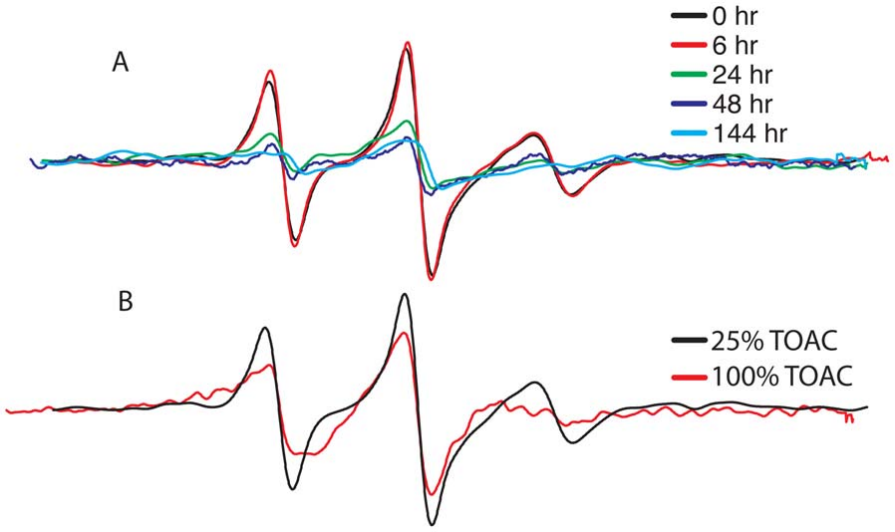
Spectral broadening due to immobilization and dipolar interaction in assemblies of Aβ. (A) Changes in the correlation time of Aβ^(26TOAC)^ with increased incubation time. To mainly observe the effects on dynamics, the spins in the Aβ preparation were diluted with wild-type peptide (Aβ_(1–40)_), so that the sample contained only 25% Aβ^(26TOAC)^. (B) Comparisons of EPR spectra of 80 µM AβO preparations containing either 25% (black line) or 100% (red line) Aβ^(26TOAC)^. Both spectra were acquired after 1 hour at room temperature.

We recently [18] reported on Aβ synthesized with a TOAC spin label positioned in the central region of the peptide, at position 26 (Aβ^(26TOAC)^). TOAC is a backbone-restricted nitroxide that offers improved detection of the dynamics arising from movement of a peptide's backbone and/or global rotational diffusion. We have previously shown that the local backbone at position 26 is sufficiently ordered such that the EPR spectrum of Aβ^(26TOAC)^ is sensitive to changes in the peptide's rate of global rotational diffusion [18]. Since oligomerization of Aβ^(26TOAC)^ will have profound effects on the rate of global rotational diffusion, this modified peptide provides direct insights into the oligomeric state of Aβ in solution. As shown in Figure 7A, there is a time-dependent broadening of the Aβ^(26TOAC)^ EPR line shape, consistent with an increasing molecular volume resulting from oligomerization. Because of the close proximity of peptides in AβO, the samples in Figure 7A contained a 25% molar fraction of Aβ^(26TOAC)^ that was spin-diluted with wild-type Aβ. Thus the increased line-broadening in these samples can be attributed to changes in spin-label correlation time, and not spin coupling. However, the strong influence of dipolar coupling in the oligomer is evident if spin dilution of Aβ^(26TOAC)^ is not carried out (Figure 7B).

### Effects of SLF observed by spin labels located within Aβ

As shown previously [6], the dynamics of Aβ containing a spin label near its N-terminus serve to indicate the disruption of AβO by active fluorene compounds. Because AβO disruption should be accompanied by increased rates of global rotational diffusion in solution, the spin labels attached to Aβ provide an additional means of observing AβO disruption by the SLF. Furthermore, if the proximity of the nitroxide moieties located on the fluorene ligand and the Aβ peptide are close enough to interact magnetically, the dipolar broadening may be evident in the composite spectrum.

**Figure 8:**
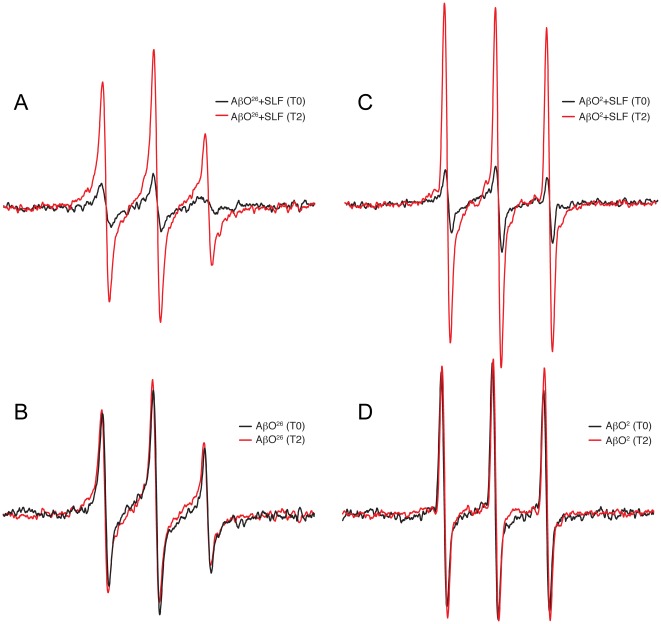
Fluorenes bind AβO and destabilize highly organized secondary structure. EPR spectra of SLF (10 µM) with single-cysteine mutant Aβ_(1–40)_ oligomers (40 µM) show that motional freedom of the mixture increased following a 2-hour incubation at room temperature (red) compared with 0 hours (black). A) and B) 26 AβO: spin labeled at 26^th^ residue C) and D) 2 AβO: spin labeled at 2^nd^ residue.

As shown in Figure 8, 2 hours after addition of the compound to AβO harboring a spin label at position 2 or 26, the spectrum greatly increases in amplitude (red trace). This demonstrates that the SLF is able to greatly disrupt the AβO after 2 hours. Since spin labels are only attached to 25% of the Aβ peptides, the level of broadening suggests the nitroxides on the oligomeric peptides can interact with more than one docked fluorene. This is supported by the observation that after complete disruption of AβO, there is a large increase in the spectral amplitude. Some increase in the amplitude is expected due to faster diffusion of monomeric Aβ [18]. Our investigation of the ability of 10 µM SLF to disrupt oligomers formed after 24 hours produced varied results that most likely reflect the heterogeneity of assembly size and structure, some of which appear to be resistant to SLF disruption.

### Interaction between the nitroxides on the SLF ligand and Aβ

**Figure 9:**
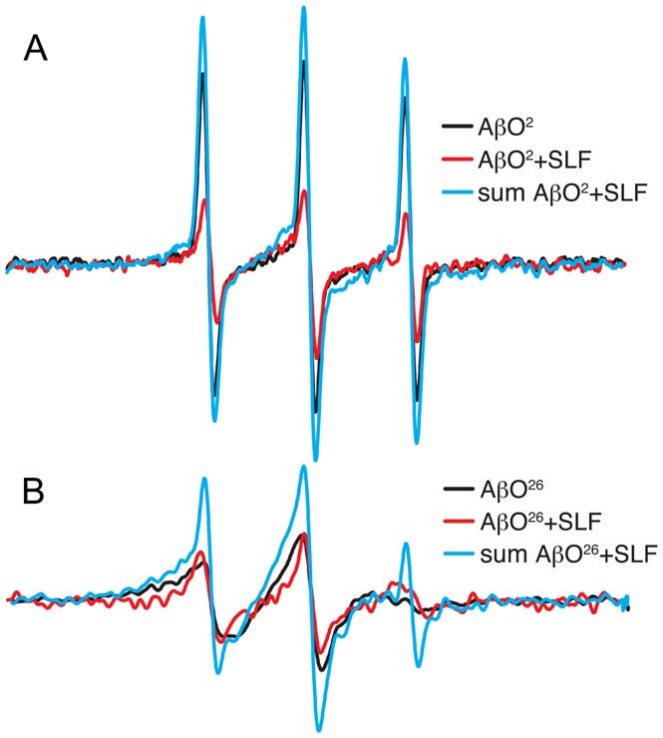
The comparison of EPR spectra from Aβ alone, an actual mixture of SLF and Aβ, and the mathematical sum of the individual EPR signals from SLF and Aβ. Data were taken for samples containing AβO spin labeled at the 2^nd^ residue (A) and for samples containing AβO spin labeled at the 26^th^ residue (B).

Figure 9 compares EPR spectra from the following conditions using Aβ spin-labeled at either position 2 or 26: a sample containing spin-labeled AβO alone, a sample containing both spin-labeled AβO and SLF, and a composite spectrum generated from the individual SLF and spin-labeled AβO spectra. From these comparisons, it is evident that both of the spin-labeled positions in AβO show at least some interaction with the nitroxide moiety on the SLF. This is based on the observation that the spectrum of the sample with both species labeled is lower in amplitude than the sample containing labeled AβO alone. Position 26 in Aβ (Fig. 9B) experiences a stronger interaction with the SLF than position 2 of Aβ (Fig. 9A). The blue trace in Figure 9A or 8B is a sum of the individual spectra of the two spin-labeled species (Aβ and SLF,) and indicates what the actual mixed sample spectrum would look like if no interaction were present. However, the observed spectrum of the mixed sample (black trace) is lower than the composite spectrum. The broadening of the mixed spectrum is slight when Aβ contains the spin label at position 2, indicating the nitroxide of SLF approaches within 2 nm of the N-terminal region. However, the broadening of the sample containing a spin label at position 26 is more substantial, demonstrating the nitroxide of SLF is in closer proximity (1.5 nm or less).

### Circular dichroism analysis of SLF influences on Aβ

**Figure 10:**
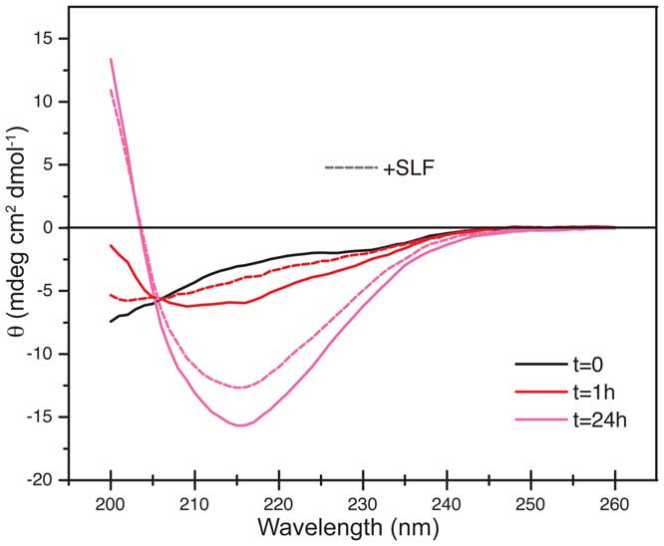
Circular dichroism analysis of secondary structural changes in Aβ. The time zero (t = 0) trace was obtained by scanning Aβ immediately after introduction into buffer. Inclusion of SLF had no significant effect on the t = 0 spectrum (not shown). Additional traces were obtained at 1, 2, 4, 6 and 24 hours. For both the control and +SLF samples, the t = 2, 4, and 6 hour traces are not shown, as these curves changed in a stepwise manner between the t = 1 and t = 24 hour time points. See Methods for additional details.

Circular dichroism (CD) spectroscopy provides a global indicator for the increase in beta-strand content that accompanies the oligomerization of the Aβ peptide [19]. To determine whether SLF affects the transition of Aβ monomers into secondary structures, CD spectra of Aβ were collected immediately after its initial introduction into aqueous buffer and then throughout a time course of 24 hours. In the absence of SLF, the CD spectra of Aβ reflect a transition from an unstructured random coil to a spectrum characteristic of beta-strand secondary structure (Fig. 10) [20]. The most substantial effect of SLF on the CD spectrum of Aβ is seen at 1 hour, where in the absence of SLF the spectrum shows a distinct beta absorption pattern. However in the presence of SLF, the peptide maintains a largely disordered structure after 1 hour. Although SLF does slow the development of beta secondary structure, by 24 hours the CD spectrum of Aβ in the presence of SLF shows strong absorption in the wavelength range that interacts most efficiently with a beta-sheet fold. As shown in Figure 10, however, the 24-hour spectra for Aβ in the presence and absence of SLF are significantly different, suggesting a unique conformation for the peptide in the presence of SLF. This is not unexpected, as the peptide's structure likely adopts a distinct fold as it incorporates into larger assemblies. As the neurotoxicity of Aβ correlates more strongly with aggregative ability than secondary structure [21], compounds that influence the former provide better candidates for intervening in the molecular pathology of AD.

### SLF scavenges free radicals

Sterically hindered alpha, alpha′-tetrasubstituted 5-membered cyclic secondary amines are sensitive to oxidation by ROS to N-oxyls. In turn, the N-oxyls can be oxidized to the N-oxoimmonium, or reduced to diamagnetic N-hydroxylamines [22], [23], [24]. A major advantage of the N-oxyl radical is that it represents a stable free radical that does *not* induce damage to DNA, proteins, lipids or sugars. Thus, this added feature of the fluorene should improve its ability to lower oxidative stress by either donating or accepting electrons with radicals (•R), such as reactive oxygen species (•ROS).

**Figure 11:**
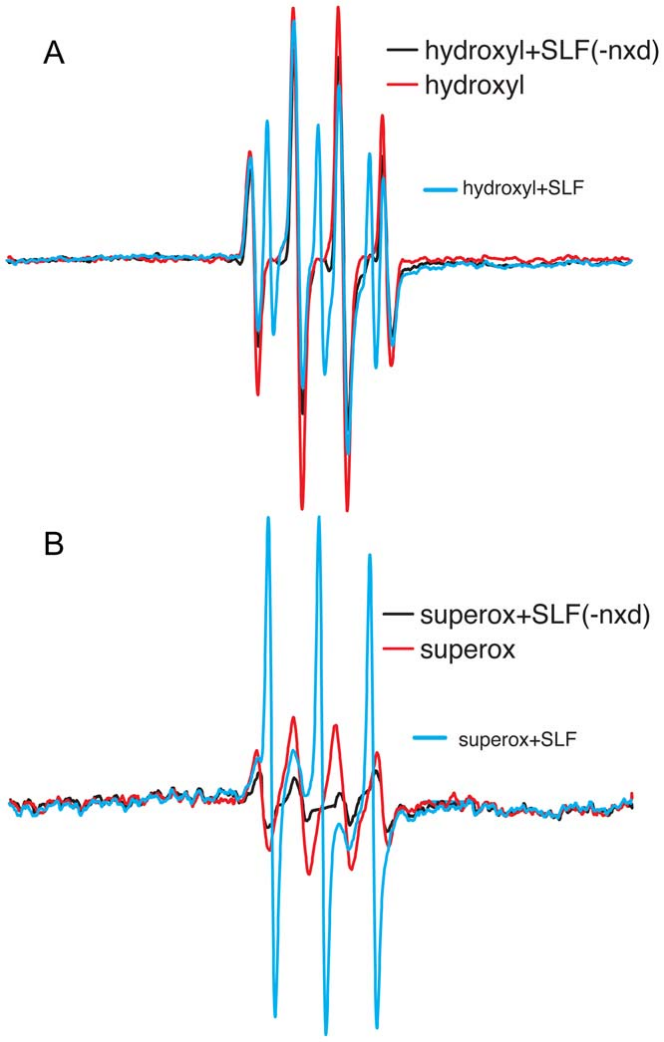
The EPR spectra of BMPO adducts with hydroxyl (A) and superoxide (B) radicals (generated via ironsulfate/H_2_O_2_ and horseradish peroxidase/H_2_O_2_, respectively) are shown in green. The spectra in the presence of SLF are shown in red, reflecting a decrease in the amount of BMPO adduct formed. Based on the difference in the spectral intensities of the generated BMPO-adduct, SLF HO-4160 is able to scavenge approximately 80% and 25% of the superoxide and hydroxyl radicals, respectively.

**Figure 12:**
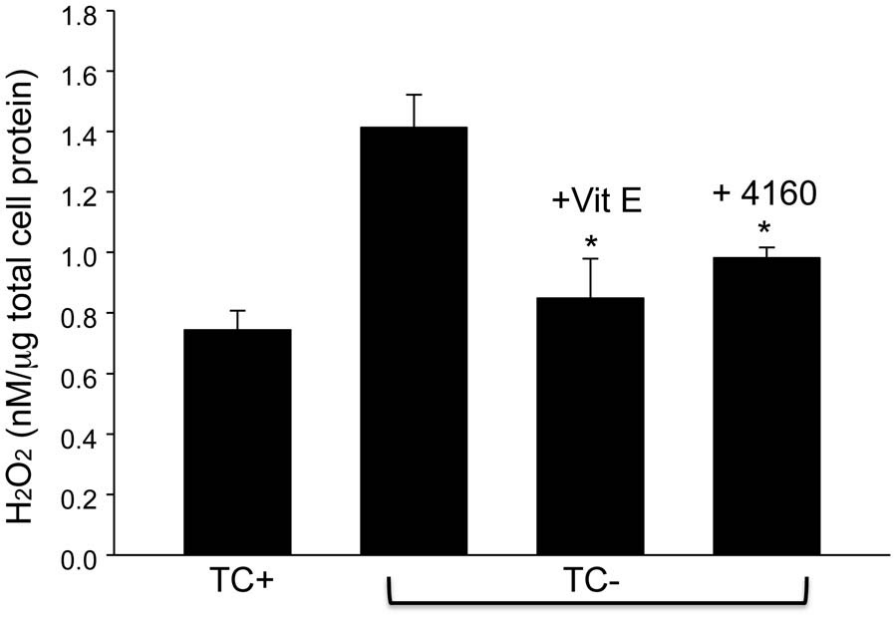
Cultured neuronal cells treated with SLF HO-4160 decrease production of hydrogen peroxide. MC65 cells were cultured in the presence (TC+) or absence (TC−) of tetracycline, a transgene suppressor of the APP fragment C99. In the absence of tetracycline, cells express the C99 fragment which is then further cleaved to form Aβ. Treatment with either the HO-4160 compound (+4160, 0.3 µM) or the antioxidant vitamin E (+Vit E, 100 µM) reduces the extracellular accumulation of hydrogen peroxide in cells expressing APP-C99. Error bars represent the SEM for N = 3. *p<0.05 TC− compared to both + Vit E and +4160.

To measure the free-radical scavenging potential of candidate compounds, spin-trapping in the presence and absence of SLF was measured by EPR spectroscopy to look for the depletion of superoxide and hydroxyl radicals. The EPR spectra of BMPO adducts with superoxide and hydroxyl radicals (generated via horseradish peroxidase/H_2_O_2_ and ironsulfate/H_2_O_2_, respectively) are shown in green (Fig. 11). The spectrum in the presence of SLF is shown in red, reflecting a decrease in the amount of BMPO adduct formed. The antioxidant activity of the SLF further extends to cellular systems. In the same MC65 neuroblastoma cell model described previously, addition of SLF attenuates the production of hydrogen peroxide in response to APP-C99 expression (−TC) compared to control cells (+TC) lacking APP-C99 (Fig. 12). The reduced hydrogen peroxide production approaches control levels and is similar to the reduced levels achieved by treatment of Aβ-generating cells with the antioxidant vitamin E.

### Summary

**Figure 13:**
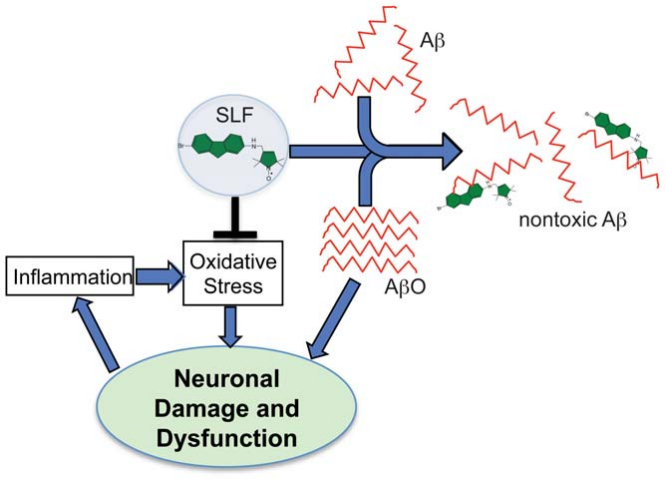
Model of action of SLFs on AβO assemblies. The illustration highlights the bifunctional properties of the SLF, including its ability to block the formation of nm particles and disrupt small oligomers, as well as its antioxidant activity. The ability of SLF to disrupt fibrils or more mature fibrillar oligomers [32] is undetermined.

We have previously shown that selected fluorene compounds can rapidly disrupt AβO in solution, and dramatically attenuate AβO toxicity *in vivo* [6]. The results reported here demonstrate how SLFs help elucidate the mechanism of fluorene action and, more importantly, that SLFs have superior potency in alleviating AβO-induced toxicity. Simulations of the SLF parent K01-162 and Aβ_(1–42)_ show preferential interaction of the compound with a hydrophobic core region of the peptide constituted by residues 17–21 [25]. Figure 13 illustrates one potential mode of interaction between AβO and SLF consistent with our findings regarding substituted fluorenes [6], where the binding of SLF to Aβ occludes a hydrophobic interface that facilitates peptide oligomerization.

## Materials and Methods

### Materials

Spin-labeled fluorene HO-4160 (7-Bromo-N —[(1-Oxyl-2,2,5,5-tetramethyl-2,5-dihydro-1H-pyrrol-3-yl)methyl]-9H-fluoren-2-amine radical) was synthesized as previously described [15]. Peptide labeled at position 26 (Aβ^(26TOAC)^) was synthesized with the TOAC nitroxide incorporated at position 26 as previously described [18]. Peptide labeled at position 2 (Aβ^(2R1)^), was first synthesized with a Cys residue substituted at position 2, and then reacted with the methanethiosulfonate spin label (MTS-SL) [26], as previously described [6]. The structures and approximate locations of each spin label are indicated in Figure 1. MC65 cells were generated by Sopher et. al. [27], and gifted to L-W.J.

### Preparation of Aβ oligomers

Solid Aβ_(1–40)_ peptide (Bachem Cat # 1194.0001, Torrance, CA) was dissolved in Hexa-Fluoro-Iso-Propanol (HFIP, Sigma, St. Louis, MO). The solution was incubated at room temperature for 1 day until the solution became clear and colorless. HFIP is a strong reducing reagent that can break hydrogen bonds and keep Aβ_(1–40)_ in the monomeric form. All the HFIP was removed by SpeedVac Concentrator (Savant, SV100H, Thermo Scientific, Waltham, MA). To generate Aβ_(1–40)_ oligomers, a 100% DMSO stock solution of 1 mM Aβ_(1–40)_ was diluted in cold PBS buffer pH 7.4 to a total concentration of 100 µM. For EPR experiments using spin-labeled Aβ, the AβO preparations contained a mixture of 25% spin-labeled peptide to 75% native Aβ_(1–40)_. This dilution of labeled peptide in the AβO sample minimizes the broadening of the EPR spectrum that arises from the dipolar interaction of spins in close proximity (<2 nm) [28].

### Cell culture models exhibiting intraneuronal AβO

The cell culture model used for these studies was the human neuroblastoma cell line (MC65) equipped with conditional expression of the carboxyl-terminal 99 residues of the amyloid-β precursor protein (APP-C99). Aβ is generated from APP-C99 after proteolysis by cellular γ-secretase. To induce cellular Aβ production, the transgene suppressor, tetracycline (TC), was removed from the media, as described previously [3]. Intracellular AβO started to accumulate as early as 4 hours after TC removal. The cytotoxicity was determined on day 3 using a colorimetric MTT [3-(4,5-dimethylthiazol-2yl)-2,5-diphenyltetrazolium bromide] assay, the results of which were comparable to data obtained using counts of viable cells based on trypan blue exclusion and the LIVE/DEAD assay (Invitrogen, Grand Island, NY). To test the fluorene compounds, the compounds were added immediately after TC removal, and the cells were maintained for 3 days without media change before the MTT assay.

### Western blotting

The preparation of cell homogenates and Western blotting were performed as previously described [29], [30].

### Immunofluorescence staining

Immunofluorescent labeling of AβO in cultured MC65 cells by the A11 anti-oligomer antibody (Chemicon-Millipore, Billerica, MA) was performed according to our previously published protocols [16].

### Atomic force microscopy (AFM)

AFM was employed to analyze the oligomer formation of wild-type AβO. All surface scans employed a Dimension 3100 Scanning Probe Microscope with a Hybrid closed-loop XYZ head and Nanoscope IVa controller (Vecco, Santa Barbara, CA). All samples were prepared on freshly-cleaved mica (Ted Pella, Redding, CA) and imaged in tapping mode in air by a phosphorous-doped silicon cantilever with a nominal spring constant of 40 N/m. Particle dimension measurements and image enhancement were performed with the Nanoscope software supplied by Veeco, version 6.14. For each measurement, an aliquot of AβO was removed from the 1 mM DMSO stock solution and diluted to 50 µM of AβO in PBS pH 7.4, and immediately spotted on freshly cleaned mica. After 2 minutes the samples were washed with 200 µL distilled water and then partially dried by compressed air and completely dried at room temperature [6].

### Thioflavin T (ThT) binding assay

Aβ_(1–40)_ (40 µM) in the presence or absence of SLF (10 µM) was incubated at room temperature for 24 hours. Aβ_(1–40)_ (20 µL) with or without SLF was incubated in 170 µL PBS buffer and 10 µL ThT (ThT stock: 0.1 mM stored in the dark at 4°C) for 15 minutes in the dark. Then ThT fluorescence was measured by a VICTOR3 Multilabel Plate Counter (PerkinElmer, Waltham, MA) spectrofluorometer at an excitation of 450 nm, with excitation and emission slit widths of 10 nm.

### Circular dichroism spectroscopy

CD measurements were performed on a Jasco J-810 spectropolarimeter equipped with a Jasco CDF-426S Peltier set to 25°C (Jasco, Easton, MD). Aβ_(1–40)_ was diluted to 40–80 µM in phosphate buffer (25 mM, pH 7.4). SLF in DMSO (final concentration 10–20 µM) or DMSO alone (final concentration 0.01–0.02%) was added to the Aβ, and the samples incubated for 0, 1, 2, 4, 6 and 24 hours at room temperature. After each incubation time point the samples were placed in a 0.1 mm quartz cuvette and, after extensive purging with nitrogen, scanned in the 200 to 260 nm region (scan speed was 20 nm/min). Averages of five scans were baseline-subtracted (25 mM phosphate with 0.01–0.02% DMSO, or 25 mM phosphate with 10–20 µM of SLF in DMSO).

### EPR spectroscopy

EPR measurements were carried out in a JEOL TE-100 X-band spectrometer fitted with a loop-gap resonator as described previously [23] (JEOL USA, Peabody, MA). SLF (10 µM) was added to the spin-labeled AβO (40 µM) at a final concentration of 32 µM for 0 and 2 hours prior to EPR measurements. Appropriate vehicle controls were used for all samples. Approximately 5 µl of the protein, at a final concentration of 32 µM was loaded into a sealed quartz capillary tube. The spectra were obtained by averaging two 2-minute scans with a sweep width of 100 G at a microwave power of 4 mW and modulation amplitude optimized to the natural line width of the attached spin probe. All the spectra were recorded at room temperature.

### Antioxidant activity

#### Measurement of superoxide and hydroxyl free radicals by EPR

The free radical scavenging activity of SLF compounds was determined by measuring the adduct levels accumulated by the spin trap BMPO. Briefly, a mixture of horseradish peroxidase (100 ng) and 0.03% hydrogen peroxide in PBS pH 7.4 was used to generate superoxide radicals. Hydroxyl radicals were generated by mixing ferrous ammonium sulfonate (0.1 mM) and hydrogen peroxidase (0.1 mM) in PBS. All EPR measurements were performed in PBS buffer pH 7.4 which contained BMPO (1 mM) in the presence or absence of SLFs at varied concentrations. The superoxide and hydroxyl radicals were measured as BMPO-OOH and BMPO-OH adducts, respectively.

#### Scavenging of peroxyl and hydroxyl radicals measured by fluorescence

Fluorescence detection was determined using the Radical Absorbance Capacity Assay (Cell Biolabs, Inc., San Diego, CA) according to the manufacturer's instructions. Briefly, the indicator is oxidized by ROS species resulting in a loss of fluorescence. Thus ROS scavenging activity is determined by the intensity of fluorescence following addition of the hydroxyl or peroxyl challenge.

#### Measurement of cellular hydrogen peroxide levels

MC65 cells were plated onto 12-well plates at 2×10^5^ cells per well in Opti-MEM with and without tetracycline (1 µg/ml). Compounds, such as HO-4160 (0.3 µM) or α-Tocopherol (referred to as vitamin E, 100 µM, Sigma, St. Louis, MO) were added to the cultures immediately after plating. Culture medium was collected after a 24-hour incubation at 37°C. Hydrogen peroxide in the conditioned medium was analyzed by the Amplex Red Hydrogen Peroxide/Peroxidase Assay kit following the instructions of the manufacturer (Invitrogen, Grand Island, NY).

## Supporting Information

Figure S1 Structures of SLF compounds [15] evaluated in Table 1.
(TIF)
