# Supplementary figures and images for: The Influence of Spin-Labeled Fluorene Compounds on the Assembly and Toxicity of the Aβ Peptide
Source: PLoS One. 2012 Apr 30;7(4):e35443. doi: 10.1371/journal.pone.0035443 (PMC3340382; doi:10.1371/journal.pone.0035443)

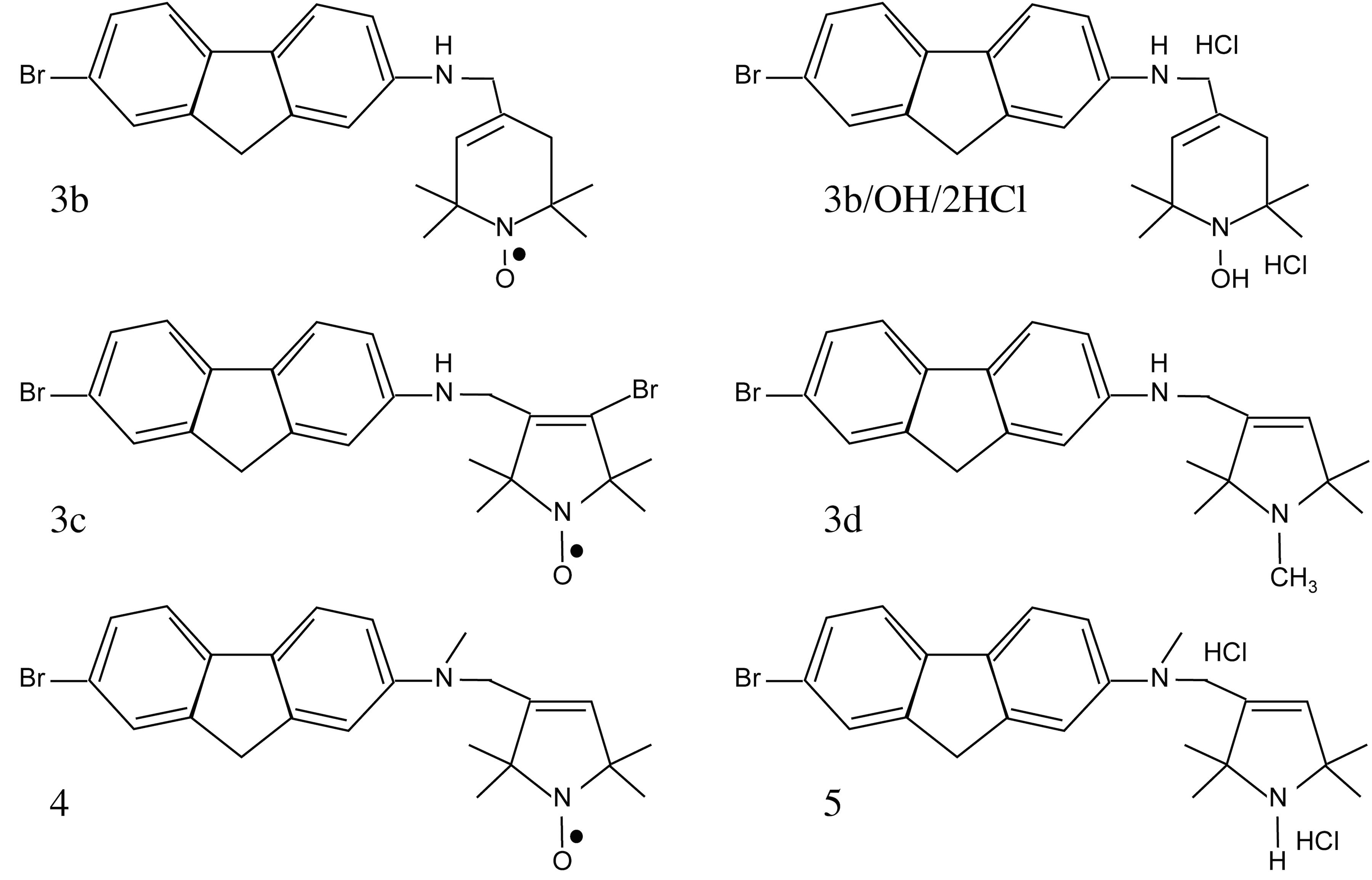

Supplement: Figure S1 — Structures of SLF compounds [15] evaluated in Table 1. (TIF) [file pone.0035443.s001.tif]
